# Polycyclic Aromatic Hydrocarbons in the Dagang Oilfield (China): Distribution, Sources, and Risk Assessment

**DOI:** 10.3390/ijerph120605775

**Published:** 2015-05-26

**Authors:** Haihua Jiao, Xiaoping Rui, Shanghua Wu, Zhihui Bai, Xuliang Zhuang, Zhanbin Huang

**Affiliations:** 1 Department of Biological Sciences and Technology, Changzhi University, Changzhi 046011, China; E-Mail: jiaohaihuaczxy@sina.com; 2 Research Center for Eco-Environmental Sciences, Chinese Academy of Sciences, Beijing 100085, China; E-Mails: shanghua1992@126.com (S.W.); xlzhuang@rcees.ac.cn (X.Z.); 3 College of Resources and Environment, University of Chinese Academy of Sciences, Beijing 100049, China; E-Mail: ruixp@ucas.ac.cn; 4 School of Chemical and Environmental Engineering, China University of Mining and Technology-Beijing, Beijing 100083, China; E-Mail: zbhuang2003@163.com

**Keywords:** polycyclic aromatic hydrocarbons (PAHs), oilfield, soil contaminant, risk assessment

## Abstract

The levels of 16 polycyclic aromatic hydrocarbons (PAHs) were investigated in 27 upper layer (0–25 cm) soil samples collected from the Dagang Oilfield (China) in April 2013 to estimate their distribution, possible sources, and potential risks posed. The total concentrations of PAHs (∑PAHs) varied between 103.6 µg·kg^−1^ and 5872 µg·kg^−1^, with a mean concentration of 919.8 µg·kg^−1^; increased concentrations were noted along a gradient from arable desert soil (mean 343.5 µg·kg^−1^), to oil well areas (mean of 627.3 µg·kg^−1^), to urban and residential zones (mean of 1856 µg·kg^−1^). Diagnostic ratios showed diverse source of PAHs, including petroleum, liquid fossil fuels, and biomass combustion sources. Combustion sources were most significant for PAHs in arable desert soils and residential zones, while petroleum sources were a significant source of PAHs in oilfield areas. Based ontheir carcinogenity, PAHs were classified as carcinogenic (B) or not classified/non-carcinogenic (NB). The total concentrations of carcinogenic PAHs (∑BPAHs) varied from 13.3 µg·kg^−1^ to 4397 µg·kg^−1^ across all samples, with a mean concentration of 594.4 µg·kg^−1^. The results suggest that oilfield soil is subject to a certain level of ecological environment risk.

## 1. Introduction

Polycyclic aromatic hydrocarbons (PAHs) are a group of persistent pollutants that are composed of two or more fused aromatic rings of carbon and hydrogen atoms, which are difficult to degrade and metabolize. PAHs are primarily byproducts of industrial processes (such as petroleum production, coke production, and transportation) and of incomplete combustion or pyrolysis of organic materials (e.g., wood, fossil fuels, and other organic materials) [1]. Once emitted, PAHs can be widely dispersed in the environment (e.g., air, water, soil, and sediment). Due to the hydrophobicity and lipophilicity of PAHs, soil could globally be acting as a sink for these. It has been documented that 90% of the total burden of PAHs reaching soil is retained in surface soil. This produces pollution hazards and imbalance of the soil ecological system [2].

PAHs have been the subject of extensive attention due to their ubiquitous presence in the environment and their potential mutagenicity, teratogenicity, and carcinogenicity [3]. Many regulations on PAH emissions have been proposed. The US Environmental Protection Agency (US EPA), for example, has listed 16 unsubstituted PAHs as priority control pollutants [4]. Subsequently, many countries, including China, placed several PAHs on a black or gray list of priority control pollutants [5,6]. Recently, studies of PAH concentrations, distribution, and risk assessment in soils have been conducted at different regional scales across the world. Xiao *et al.*, for example, estimated the distribution and possible sources of PAHs in superficial soils of the Pearl River Delta [7]. Agarwal *et al.*, analyzed the contents of 16 PAHs in surface soil in various agricultural sites in Delhi [8]. Maliszewska-Kordybach *et al.*, provided comprehensive information on the level of PAH contamination in arable soils in Poland [9]. Sverdrup *et al.*, studied the ecotoxicity of PAHs in soil [10]. However, there has been less research on the concentration, distribution, and possible sources of PAHs in oilfield soils, as compared to urban and agricultural soils.

Dagang Oilfield is located between 38°40′17″ N and 39°0′0″ N and between 117°20′0″ E and 118°0′0″ E, with an elevation of 3.5 m above mean sea level, near Bohai Sea, southeast of Tianjin city in North China. Oil exploitation began in 1964. The production capacity of crude oil is currently about 5 million tons per year. The area is characterized by a petrochemical complex, intensive traffic and a population of more than 200 thousand. Recently, several studies indicated that petroleum hydrocarbon was widespread in the soil of the oilfield and its surrounding zone [11]. However, little is known about the PAH contamination in the soil of this area.

This study thus aimed to: (1) determine the level and distribution of PAH contamination in the soil of Dagang Oilfield; (2) explore the possible sources of PAHs in soils along a gradient of different land uses in the Dagang Oilfield region; and (3) assess the potential health risk posed to humans from long-term exposure to oilfield soils. It is hoped that the results of this study will provide a better understanding of the distribution patterns of soil PAHs in relation to their various origins and locations; the study also provides useful information about the health risks associated with PAH exposure, which may help in pollution treatment and in planning agricultural production in the region. 

## 2. Experimental Section

### 2.1. Sampling

**Figure 1:**
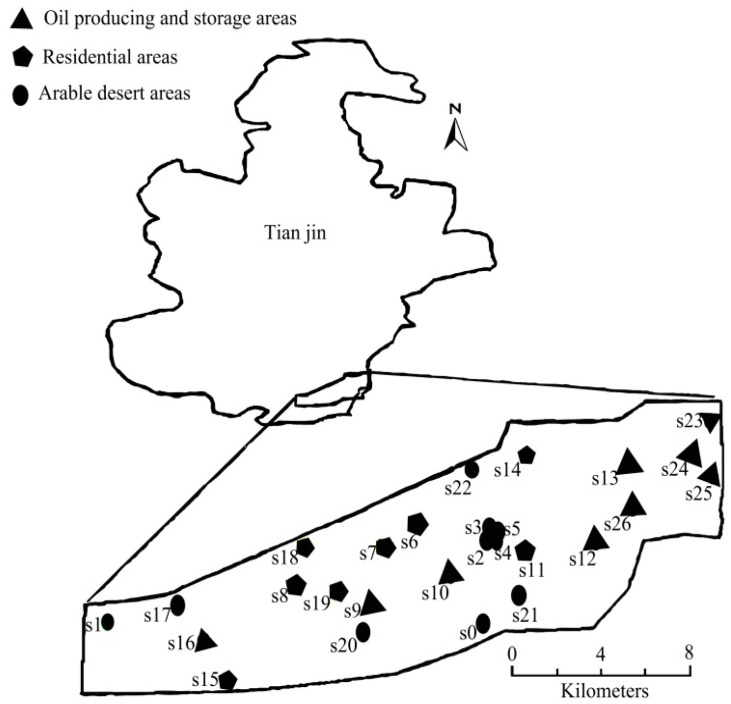
Map of study area and location of sampling sites.

In April 2013, three sampling areas were selected for the measurement of soil PAH concentrations in the Dagang Oilfield: Oil producing and storage areas (OW), a residential area (RD), and the arable desert area (AD) which is arable land but not used as farmland. There were 27 composite samples collected from the three areas. The location of the sampling sites is shown in Figure 1. Samples S0–S5, S17, and S20–S22 were collected from AD areas. Samples S9–S10, S12–S13, S16, S23–S26 were collected from OW areas. Samples S6–S8, S11, S14–S15, and S18–S19 were collected from RD areas. Surface soil samples (0–25 cm depth) were taken with a stainless steel soil auger after removal of uppermost plant cover. Samples were dried in the laboratory, and twigs and stones were removed. Soil samples were sieved through a 1 mm sieve and representative samples were obtained after coning and quartering.

### 2.2. Reagents and Instruments

A standard mixture containing 16PAHs: naphthalene (Nap), acenaphthene (Ace), acenaphthylene (Any), fluorene (Flu), phenanthrene (Phe), anthracene (Ant), fluoranthene (Fla), pyrene (Pyr), benz [а] anthracene (Bаa), chrysene (Chr), benzo [b] fluoranthene (Bbf), benzo [k] fluoranthene (BkF), benzo [а] pyrsne (Bаp), dibenz [а ,h] anthracene (Daa), benzo [g, h ,i] perylene (Bghip), indeno [1, 2 ,3-cd] pyrene (Ind), was purchased from Supelco (Bellefonte, PA, USA). Deuterium labeled surrogate standards [naphthalene-d8 (Nap-d8), acenaphthene-d8 (Ace-d8), phenanthrene-d10 (Phe-d10), fluoranthene-d10 (Fla-d10), pyrene-d10 (Pyr-d10), benzo [a] anthracene-d12 (Baa-d12), benzo [a] pyrene-d12 (Bap-d12), benzo [g, h, i] perylene-d12 (Bghip-d12) and perylene-d12 (Per-d12) (Dr. Ehrenstorfer, Germany) were used for the calculation of the extraction efficiencies, purchased from Ultra Scientific Analytical Solutions (North Kingstown, USA). All solvents: acetone, dichloromethane, tri-chloromethane, *etc.*, used for sample processing and analysis were of analytical grade (AR) and n-hexane was of high performance liquid chromatography (HPLC) grade (GR) (National medicine pharmaceutical factory). Silicagel (80–100 mesh, MerckKGaA, Darmstadt, Germany) was activated approximately for 24 h at100°C; granular anhydrous sodium sulfate was baked at 130°C for10 h before use. Agilent 6890 N gas chromatography-5973 mass spectrometer (GC/MS) system (USA, Agilent Technologies Inc., Clara, CA, USA) was used for analysis.

### 2.3. Methodology for Determination of PAHs

Soil samples were extracted using the modified methods of the US EPA ultrasonic oscillation extraction method 3540 [12]. Soil samples (10 g) were extracted three times in a conical flask with 30 mL mixture solvents (acetone: dichloromethane = 1:1) for 15 min at 30 °C, using ultrasonic agitation (ultrasonic agitation -SB 5200 DT, Ningbo Xingzhi Biological Technology Co. Ltd., Ningbo, China) at a frequency of 40 kHz. The extract was filtered through a funnel filled with anhydrous sodium sulfate, and then the soil extract was concentrated to 0.5 mL with a gentle stream of nitrogen (purity 99.999%) at 40 °C. The extract was cleaned up using a column (15 cm length × 10 mm id) filled with anhydrous sodium sulfate and silica gel. The column was made up of 10.0 g silica gel (activeness for 24 h at 100 °C) in the lower part and 5.0 g anhydrous sodium sulfate (dryness for 10 h at 200 °C) in the upper part. The column was prewashed with 30 mL acetone and 30 mL dichloromethane immediately. The extract was passed through the column, eluted with 60 mL mix of n-hexane and dichloromethane (volume (v:v), 1:1) at a flow rate of 0.5 ml·min^−1^. The elution which contains PAHs was collected and concentrated to 200 μL then reduced into n-hexane and the final volume was adjusted to approximately 1mL under a gentle stream of nitrogen prior to GC-MS analysis.

The total PAH content of the soil samples was determined using GC/MS system. The column oven temperature was initially held at 60 °C for 2 min, then increased to 230 °C at a rate of 8 °C·min^−1^ and held for 2 min, then increased to 290 °C at a rate of 2 °C·min^−1^ and held for 2 min, and finally increased to 300 °C at a rate of 5 °C·min^−1^ and held for 12 min. The injector temperature was maintained at 300 °C and highly pure helium (99.999%) as the carrier gas was maintained at a constant flow rate of 1 mL·min^−1^. A certified solution of a standard mixture containing 16 PAHs was used for the calibration of the GC-MS system. Deuterium labeled surrogate standards were used for the calculation of the extraction efficiencies.

### 2.4. Quality Control

Analytical methods were checked for precision and accuracy. All samples were analyzed in triplicate. For quality control, the analysis also included processing of a procedural blank (solvent) and a spiked blank (16 PAH standards spiked into solvent).The recovery efficiency was checked by analyzing soil samples spiked with a known amount of the PAH standard (matrix spike of 100 µg·kg^−1^ for each PAH). Limits of detection (LODs) for the 16 PAHs were calculated as the standard deviation of the six concentrations of samples. LODs for the 16 PAHs were measured from 0.12 µg·kg^−1^ (Nap) to 0.62 µg·kg^−^^1^ (Bghip). Matrix spike experiment recoveries of certified reference materials ranged from (87% ± 3%) to (98% ± 4%) for the 16 PAHs. The relative standard deviation for individual PAHs identified in triplicate samples were all <10%.Blank sample measurements were conducted in orderto be certain that other sample components would not affect absorbance measurements for the priority PAHs.

### 2.5. Statistics

Statistical analyses were performed using SPSS17.0 software. The precision of the results was expressed based on standard deviation values.

## 3. Results and Discussion

### 3.1. PAHs Concentrations in Soil Samples

Table 1 gives the concentrations of 16 PAHs (ΣPAHs) listed within the US EPA’s priority list in soils in the Dagang Oilfield. Among them, 13 PAHs were detectable in most of the samples. The ΣPAHs varied between 103.6 µg·kg^−1^ and 5872 µg·kg^−1^. According to the classification system suggested by Maliszewska-Kordybach [13], a ΣPAHs soil concentration below 200 µg·kg^−1^ indicates no contamination, a concentration of 200–600 µg·kg^−1^ represents weak contamination, and a soil concentration of 600–1000 µg·kg^−1^ represents moderate contamination. Concentrations over 1000 µg·kg^−1^ would be indicative of severe contamination. Sampling sites can thus be divided into four groups. Severe contamination (ΣPAHs ≥ 1000 µg·kg^−1^) was observed in S19, S11, S15, S25, S26 and S8, with these sites mainly located in RD areas. Moderate pollution (600 μg·g^−1^ ≤ ∑PAHs < 1000 μg·kg^−1^) was observed in S4, S12, S10, S14, S6, and S13, with these sites distributed in RD and OW areas. The slightly polluted sites (200 ≤ μg·kg^−^^1^ ∑PAHs < 600 μg·kg^−^^1^) were S0, S2, S5, S7, S9, S18, S21, S22, S23 and S24, with these mainly located in AD and OW areas. No contamination (∑PAHs < 200 μg·kg^−^^1^) was recorded from S1, S3, S16, S17, and S20, with these sites also mainly located in AD and OW areas.

**Table 1:** Distributions of different PAHs in sample soils from three areas: Urban and residential areas (RD), around oil wells (OW), and in the arable desert soil area (AD) (μg·kg^−1^), not detectable (ND).

**PAH**	**Arable Desert Soil Area (AD)**									
	**S0**	**S1**	**S2**	**S3**	**S4**	**S5**	**S17**	**S20**	**S21**	**S22**
Nap	283.6	2.26	476.0	37.41	505.6	478.5	ND	ND	ND	ND
Baa	ND	ND	7.11	6.99	ND	ND	0.49	0.9	0.83	0.46
BkF	4.13	ND	ND	ND	ND	4.13	3.09	3.13	4.05	2.39
BaP	4.98	ND	5.55	ND	ND	5.6	5.09	5.00	5.16	4.65
Ind	15.13	15	ND	ND	ND	15.1	15.11	6.13	15.82	7.13
Ace	ND	ND	ND	ND	ND	ND	ND	ND	0.21	ND
Flu	8.65	35.27	3.39	5.59	9.19	6.53	15.02	14.53	24.88	16.58
Phe	22.82	34.22	ND	33.88	52.36	25.03	54.27	108.70	218.7	196.2
Ant	20.26	0.21	ND	0.19	0.21	0.22	ND	ND	ND	ND
Fla	9.43	12.23	9.46	34.72	32.21	17.03	10.37	21.85	93.24	25.42
Pyr	3.52	3.60	3.6	24.31	50.0	3.6	6.86	28.88	116.2	27.93
Bghip	0.30	0.80	0.20	0.10	0.80	1.10	7.19	7.46	13.93	8.46
∑PAHs	372.8	103.6	505.3	143.2	650.3	556.8	117.5	196.6	492.9	289.2
∑BPAHs	307.8	17.3	488.7	44.4	505.6	503.3	23.8	15.2	25.9	14.6
**PAHs**	**Around oil well areas (OW)**									
	**S9**	**S10**	**S12**	**S13**	**S16**	**S23**	**S24**	**S25**	**S26**	**-**
Nap	489.8	689.8	738.5	471.3	ND	ND	ND	ND	20.85	-
Chr	ND	ND	ND	ND	ND	ND	ND	199.6	203.7	-
Baa	ND	ND	7.12	7.1	0.68	0.52	0.47	73.42	0.89	-
BkF	ND	4.15	ND	4.14	2.02	3.13	3.6	92.78	4.05	-
BaP	ND	0	5.58	6.03	5.47	4.54	5.62	23.85	23.85	-
Ind	ND	16.0	ND	ND	7.13	5.13	6.13	15.13	15.82	-
Ace	ND	ND	ND	ND	ND	ND	ND	ND	ND	-
Flu	9.97	3.39	7.44	5.21	15.78	11.67	13.82	18.22	129.1	-
Phe	52.33	ND	23.13	26.10	53.10	140.3	90.59	268.4	512.5	-
Ant	0.23	ND	0.25	0.27	ND	ND	ND	ND	ND	-
Fla	9.43	9.41	9.43	18.55	18.77	19.88	16.05	157.05	48.11	-
Pyr	24.31	3.59	17.63	55.97	19.38	25.11	198.7	175.6	46.80	-
Bghip	7.70	9.20	8.30	6.90	6.93	6.77	5.66	189.4	17.4	-
∑PAHs	593.8	735.6	817.4	601.6	129.3	217.0	340.6	1214	1029	-
∑BPAHs	489.8	710.1	751.2	488.6	15.30	13.32	15.82	404.7	269.2	-
**PAHs**	**Resident (RD)**									
	**S6**	**S7**	**S8**	**S11**	**S14**	**S15**	**S18**	**S19**	**-**	**-**
Nap	517.3	460.5	571.4	602.8	548.7	2.75	0.37	26.73	-	-
Chr	ND	ND	52.96	288.9	ND	ND	ND	2966.1	-	-
Baa	ND	7.01	47.70	397.8	7.13	0.86	0.91	0.87	-	-
BkF	4.10	3.99	4.13	495.2	4.10	4.13	4.97	3.97	-	-
BaP	5.70	5.71	0.00	427.1	ND	5.65	4.95	1384.1	-	-
Ind	15.13	ND	57.70	410.9	ND	1579.8	6.26	15.40	-	-
Ace	ND	ND	ND	15.02	ND	ND	ND	2.86	-	-
Flu	3.39	7.01	11.50	28.36	11.07	45.01	32.46	168.5	-	-
Phe	28.03	15.12	89.62	268.4	43.67	269.8	164.9	374.7	-	-
Ant	0.25	0.20	0.22	39.08	0.26	ND	ND	ND	-	-
Fla	39.45	55.04	89.02	411.9	6.28	54.62	71.17	806.5	-	-
Pyr	3.59	12.80	87.65	379.9	34.03	41.60	31.52	105.91	-	-
Bghip	1.20	5.20	8.60	17.05	5.89	17.46	17.61	16.19	-	-
∑PAHs	618.1	572.5	1021	3783	661.1	2022	335.1	5872	-	-
∑BPAHs	542.2	477.2	733.9	2623	559.9	1593	17.46	4397	-	-

### 3.2. Composition and Profiles of PAHs in Individual Sampling Sites

**Figure 2:**
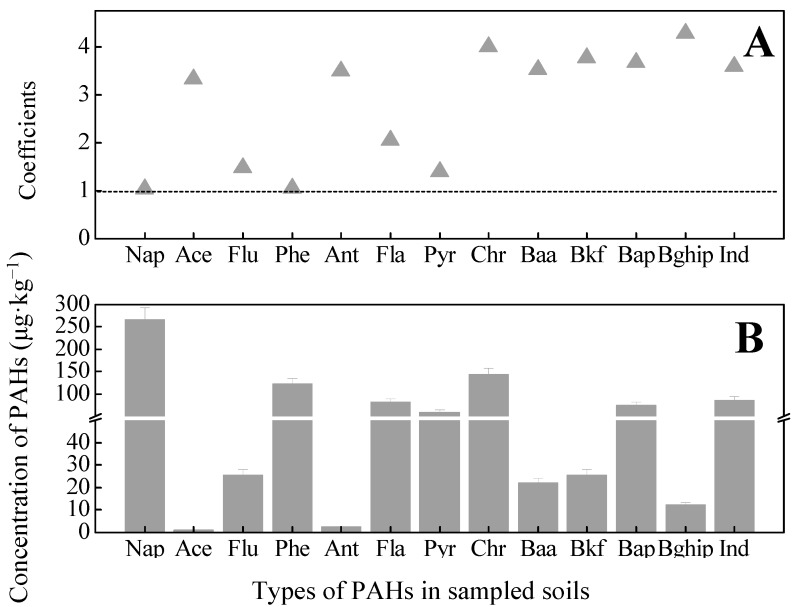
The variable coefficients and mean concentrations (µg·kg^−1^) of individual PAHs in the all sampling soils. Notes: (**A**): shows the coefficients of individual PAHs in all the sampling soils; (**B**): shows the mean concentrations of individual PAHs in all the sampling site soils; Nap-naphthalene, Ace-acenaphtene, Flu-fluorene, Phe-phenanthrene, Ant-anthracene, Fla-fluoranthene, Pyr-pyrene, Baa-benzo [a] anthracene, Chr-chrysene, Bkf-benzo [k] fluoroanthene, BaP-benzo [a] pyrene , Bghip-Benzo [g, h, i] perylene, and Ind-indeno [1, 2, 3-cd] pyrene.

The variable coefficients (ratio of standard deviation/mean) of different PAHs obtained in all sample sites were > 1.0 (Figure 2A). Thirteen PAHs listed within the US EPA’s priority list were detectable in the sampling sites. This result indicates that the types and concentrations of PAHs were significantly distinct across sample site soils. Mean concentrations of individual PAHs are presented in Figure 2B. The ratio of detectable PAHs in all sampling sites was as follows: 100% for Flu, Fla, and Pyr; 69% for Nap and Ind; 77% for Baa and Bkf; 92%, 73%, 50%, 46%, 19%, and 15% for Phe, BaP, Ant, Bghip, Chr, and Ace, respectively. The results showed that the relative abundance of PAHs with different molecular structures varies significantly across sampling site soils, due to their different locations in the oilfield.

**Figure 3:**
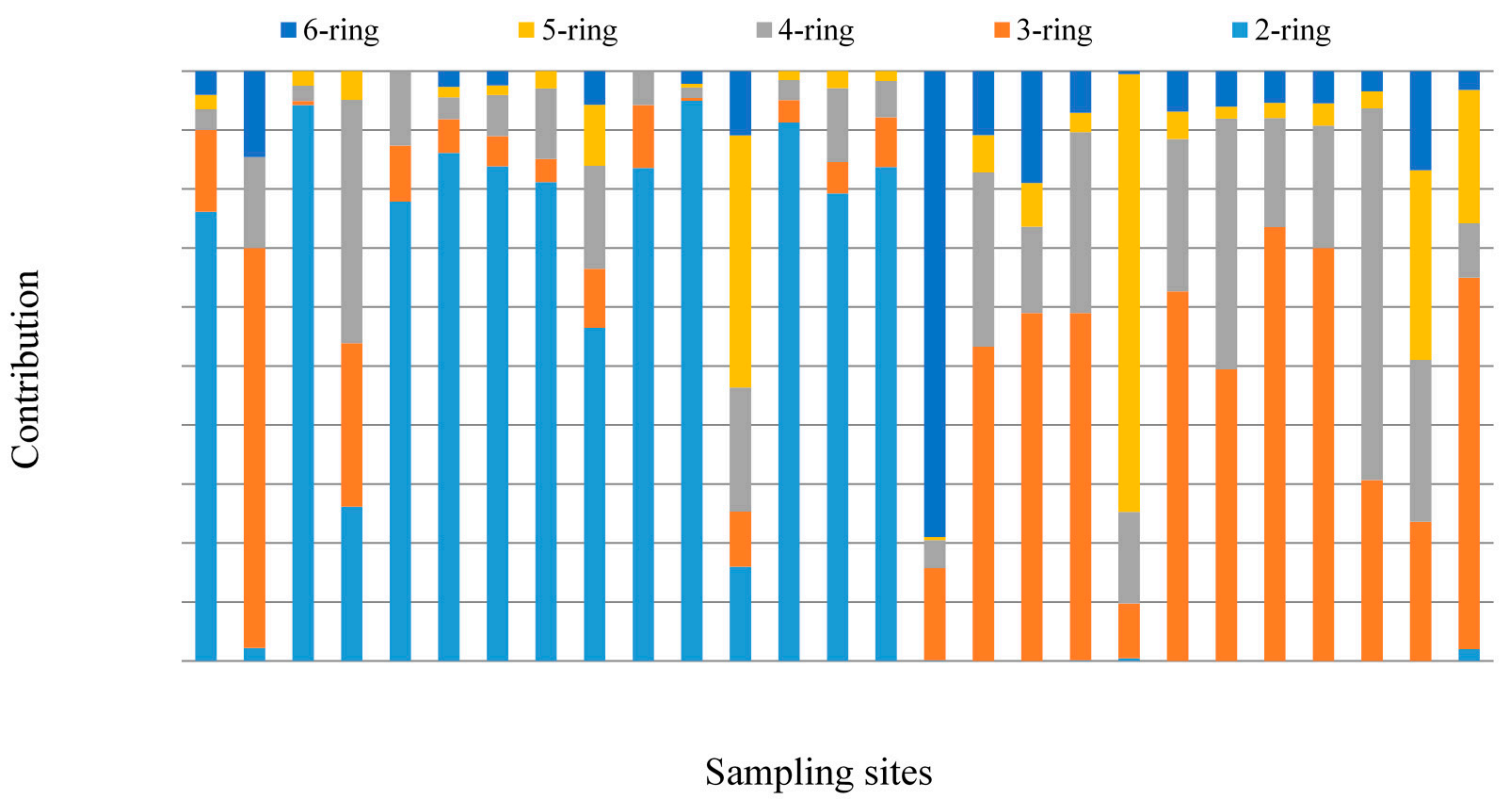
Composition of PAHs in different sampling site soils of the oilfield (2-ring: Nap; 3-ring: Ace, Flu, Phe, and Ant; 4-ring: Fla, Pyr, Baa, and Chr; 5-ring: BkF, BaP; 6-ring: Bghip, Ind).

Kuang *et al.*, reported that the major pollutants in agricultural soils around oil sludge plants in the Zhongyuan Oilfield were mainly 2-ring, 3-ring and4-ring PAHs [14]. Based on the number of aromatic rings, the PAHs detected in sampling sites soils can be subdivided into five groups: e.g., 2-ring, 3-ring, 4-ring, 5-ring, and 6-ring PAHs. The profiles of PAHs in individual sample sites are shown in Figure 3. PAHs with 2 and 3 rings were predominant in study area soils. The compositions of 2-ring PAHs ranged from ND to 95.0%, with a mean of 38.4%, while the compositions of 3-ring PAHs ranged between 0.47% and 43%, with a mean of 27.8%. Percentage compositions of 4-ring, 5-ring, and 6-ring PAHs were 1.79%–63.0% (mean of 17.4%), ND–74.2% (mean of 8.8%), and ND–79.0% (mean of 7.6%), respectively.

### 3.3. Analysis of Potential Contamination Sources

#### 3.3.1. Isomer Ratios

Although the use of PAH diagnostic ratios has been criticized in the past [15,16,17], it has been used widely and therefore, in the present study it is used as an indicative source apportionment tool. The ratio of low molecular weight PAHs (LMW-PAHs, ring ≤ 3 PAHs) to high molecular weight PAHs (HMW-PAHs, ring ≥ 4) [18], as well as several PAH isomer ratios, have been widely used to identify possible sources of PAHs in environmental samples. These include Fla/Pyr, Fla/ (Fla + Pyr), Baa/ (Baa + Chr), and Ind/ (Ind + Bghip) [19,20]. PAHs originating from petrogenic sources are dominated by 2-ring and 3-ring PAHs, while HMW-PAHs with ≥4-rings are indicative of pyrogenic origin [21]. Yunker *et al.*, suggested that a Fla/ (Fla + Pyr) ratio of <0.4 indicates a petroleum source, while a ratio of >0.5 indicates a pyrogenic source, *i.e.*, combustion source of biomass (grass, wood, or coal combustion). A Fla/ (Fla + Pyr) ratio of 0.4–0.5 implies petroleum combustion, e.g., liquid fossil fuel, vehicle exhaust, and crude oil. Baa/ (Baa + Chr) ratios of <0.2 indicate petrogenic (petroleum input) origin, while a ratio >0.35 implies pyrogenic (combustion) origin. If the Baa/ (Baa + Chr) ratio is between 0.2 and 0.35, this would suggest mixed origin [22]. A ratio of Ind/ (Ind+Bghip) <0.2 implies a petroleum source, a ratio of >0.5 indicates biomass and coal combustion sources, and 0.2 < ratio < 0.5 suggests a liquid fossil fuel combustion source [23]. Moreover, other ratios have been used to identify PAHs with biomass or petroleum combustion origins. These include the concentration ratio of Fla/Pyr, used as a standard to determine the sources of PAHs by many researchers. Sicre *et al.*, have reported that a Fla/Pyr ratio of >1 is indicative of PAHs of fossil fuel combustion origin, while a Fla/Pyr ratio of < 1 is indicative of PAHs of petrogenic origin [24].

**Figure 4:**
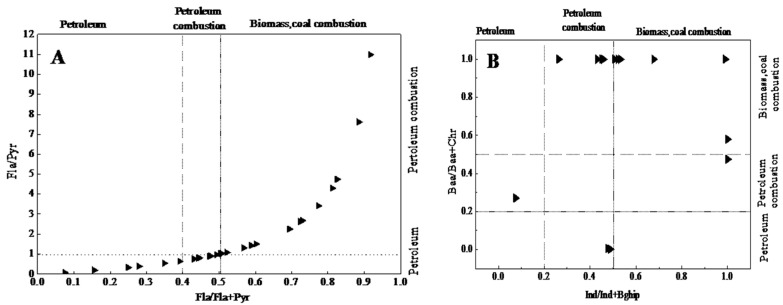
PAHs cross plots for ratios of Fla/Pyr *vs.* Fla/(Fla + Pyr); Baa/(Baa + Chr) *vs.* Ind/(Ind + Bghip). Notes: (**A**): shows the cross plots for ratios of Fla/Pyr *vs.* Fla/ (Fla + Pyr); (**B**): shows the cross plots for ratios of Baa/ (Baa + Chr) vs. Ind/(Ind + Bghip); Fla-fluoranthene, Pyr-pyrene, Chr-chrysene, Baa-benzo [a] anthracene, Bghip- Benzo [g, h, i] perylene, and Ind-indeno [1, 2, 3-cd] pyrene.

Figure 4 is a plot of the ratios of Fla/ (Fla + Pyr), Baa/ (Baa + Chry), Ind/ (Ind+Bghip), and Fla/Pyr. In this study, the ratios of Fla/ (Fla + Pyr) varied from 0.16 to 0.92 (Figure 4A), Baa/ (Baa + Chr) ratios were >0.35 or <0.20 (Figure 4B) in all sampling sites, and ratios of Fla/Pyr varied between 0.18 and 10.94. A Fla/ (Fla + Pyr) ratio of <0.40 was found at S4, S9, S12, S13, and S14; a Fla/ (Fla + Pyr) ratio of >0.50 was found at S0–S3, S5–S8, and S10; at sampling sites S9, S12, S16, S20–S23, the ratio varied between 0.4 and 0.5; a Baa/(Baa + Chr) ratio of <0.2 was noted at S0, S1, S4–S6 and S9–S10 while a ratio of >0.35 was noted at S1–S3, S7–S8, and S11–S13; a Fla/Pyr ratio of <1 was noted at S2, S4, S9–S10, and S12–S14 and a ratio of >1 was found at S0, S1, S3, S5–S8, and S11; all samples from AD soils with Ind/ (Ind + BghiP) ratios above 0.50 and 62.5% and 44.4% of the sample sites were above 0.50 in RD and OW area soils, respectively. Additionally, the ratio of LMW-PAHs to HMW-PAHs varied from 0.05 to 0.81, with a mean of 0.27 across all sampling sites. The study shows that the sources of PAHs in different sampling sites can be attributed to their location. In OW sites, petroleum leaks could be a major source of PAHs. In AD sites, petroleum and biomass combustion could be the main sources. Coal combustion and traffic emission are the main contributors of PAHs in RD area sampling site soils. 

**Figure 5:**
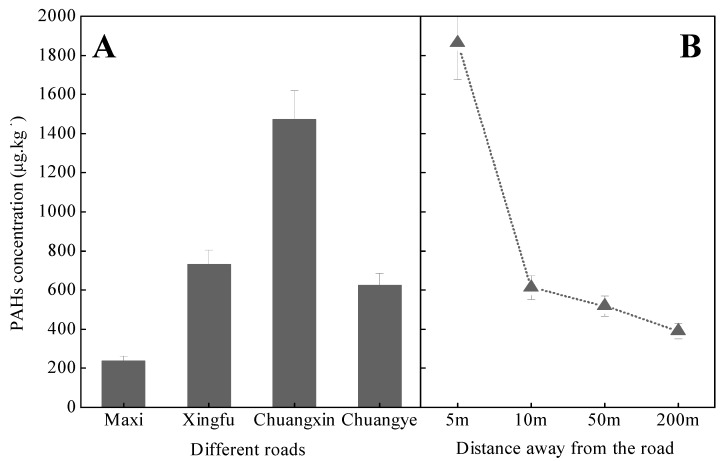
Changes of the means concentrations (µg·kg^−1^) of ∑PAH in the site soils along the different roads. Notes: (**A**): shows the means concentrations of ∑PAH for different sites along the different road; (**B**): shows the change of the mean concentrations of ∑PAH along a gradient with distance (D) from a road at D < 5m, 5m < D < 10m, 10m < D < 50m, and 50m < D < 200m, respectively.

Figure 5A shows that the distinction in the means concentrations of ∑PAH for different sites along the Maxi Road, Xingfu Road, Chuangxin Road, and Changye Road, respectively. Figure 5B reveals that the mean concentrations of ∑PAH appears to decrease along a gradient with distance (D) from a road at D < 5m, 5m < D < 10m, 10m < D < 50m, and 50m < D < 200m, respectively. Analysis of the variance trend of ∑PAHs indicates that ∑PAHs was significantly (*p*< 0.05) higher at D< 5 m than at D > 10m sampling sites, which can be ascribed to traffic activity. Many studies have reported that PAHs are primarily emitted to the atmosphere, and after transport over short and long distances in both gaseous and particulate forms, they accumulate in soils after dry and wet atmospheric deposition [25]. The result thus reflects the known relationship between PAH loadings and distance from emission sources.

#### 3.3.2. Relationship to Anthropogenic Activities

Table 1 shows the distributions of ΣPAHs in Dagang Oilfield soils. It reveals a decreasing gradient along RD-OW-AD areas. Soil PAHs mean concentrations are 1856 µg·kg^−1^ 627.3 µg·kg^−1^ and 342.5 µg·kg^−1^, respectively. The mean concentrations of PAHs in RD soils, compared with concentrations in other urban soils from all over the world, show similar levels, e.g., the Pearl River Delta (960.0–1800 µg·kg^−1^) [26], Beijing (mean of 1228 µg·kg^−1^) [27], Shanghai residential areas (mean of 1700 μg·kg^−1^) [28], Lisbon, Portugal (1544 μg·kg^−1^), and Torino, Italy (1990 μg·kg^−1^) [29], but higher concentrations than Hong Kong (140.0 μg·kg^−1^) and Harbin, China (837.0 μg·kg^−1^) [30,31].The concentrations of PAHs in OW areas were similar to soils collected from the vicinity of chemical and petrochemical industries in Tarragona County, Spain (112–1002μg·kg^−1^) [32]. The concentrations of PAHs in AD were similar to those in agricultural soils in suburban areas around Hong Kong (422.0 µg·kg^−1^) [30]. This indicates that the major sources of soil PAHs were anthropogenic activities.

**Figure 6:**
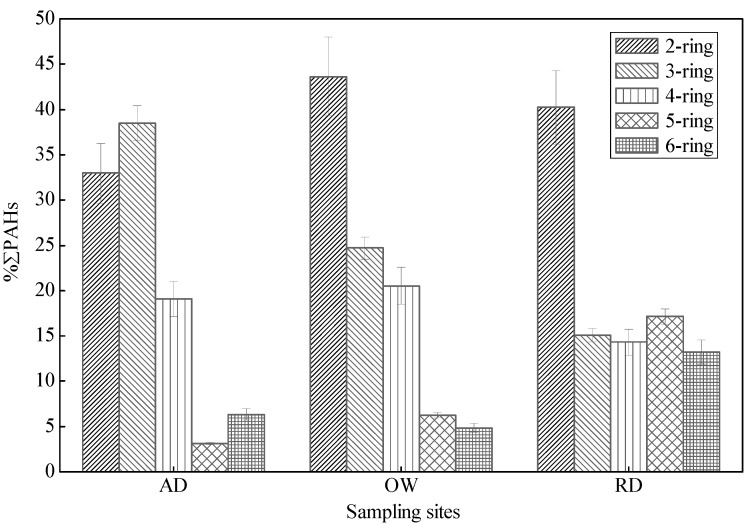
Composition profiles of PAHs in arable desert area (AD), oil producing and storage areas (OW), and residential area (RD) soil samples.

Figure 6 shows the percentages of individual PAHs to total PAH concentrations in obtained soil samples. It reveals that low molecular weight PAHs (LMW-PAHs, ring ≤ 3 PAHs) were dominant in the oilfield areas. The percentages of LMW-PAHs accounted for 71.5%, 68.4%, and 55.3% decreased along an AD-OW-RD gradient. However, the percentage of total high molecular weight PAHs (HMW-PAHs, ring ≥ 4) increased along the AD-OW-RD gradient, ranged from 28.6%–31.6%–44.7%. Low and high molecular weight PAHs had different levels of dominance in AD, OW, and RD soils, implying distinct emission sources in different areas.

Based on the results, it can be concluded that the concentrations of PAHs in oilfield soils represent a moderate level of contamination, with variations across sampling site soils. Contamination increases along an AD-OW-RD gradient. There are various sources of PAHs, such as, petroleum leaks resulting from oil exploration and processing, and incomplete combustion or pyrolysis of organic material (e.g., traffic, industry, and domestic heating, and processes of carbonaceous matter incineration). Most notable are byproducts resulting from anthropogenic activities, e.g., traffic, leading to increasing PAH levels in soils along roads. In general, sources of PAHs in the oilfield soil environment can be mainly attributed to vehicular emissions, oil exploration, oil refinery, and petroleum storage, and residential heating, among other sources.

### 3.4. Assessment of Soil Toxicity

According to the carcinogenic classification of PAHs by the US EPA and by the International Agency for Research on Cancer (IARCA), the 16 priority PAHs considered in this study can be divided into two groups: B (carcinogenic) and NB (not classified or non-carcinogenic). In this study, six PAHs considered carcinogenic or possible/probable carcinogens (*i.e.*, BaP, Nap, Baa, Chr, BkF, and Ind) accounted for 46.2% of total species of PAHs and were detected in all sampling sites. The remaining PAHs were classified as NB. In all 27 sample sites, total concentrations of carcinogenic PAHs (∑BPAHs) varied from 13.3 μg·kg^−1^ to 4397 μg·kg^−1^, with a mean concentration of 594.4 μg·kg^−1^ (Table 1).

**Figure 7:**
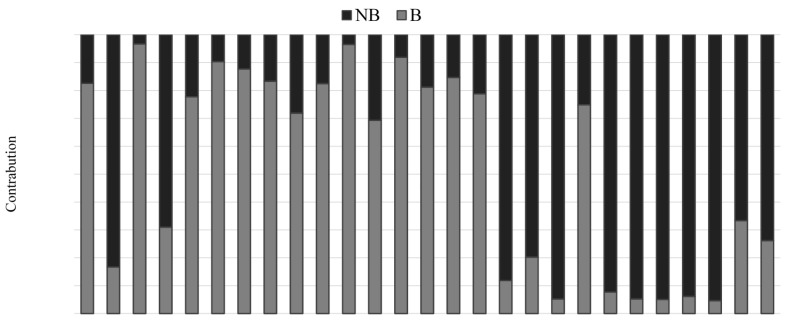
Contribution of carcinogenic PAH compounds to total PAHs in different sites. B: Classified carcinogenic PAHs; NB: Not classified and non-carcinogenic PAHs.

The distributions of B and NB PAHs in the 27 sample sites are shown in Figure 7. The percentage of BPAHs to ∑PAHs observed ranged from 5.06% to 96.7%, with a mean of 66.9% across all sampling sites and increased along the gradient AD (40.5%)-OW (48.3%)-RD (69.5%).

This study utilized toxic equivalency factors (TEFs) to estimate the exposure risks posed by individual and total PAHs to human health. The toxicities of PAHs in sampling sites were evaluated using the relative toxicity value (TEF) of each individual PAH compound, and expressed as its BaP equivalent concentration (BaPeq). TEFs of PAHs have been previously proposed by the US EPA and by Nisbet and LaGoy [33,34]. Previously indicated TEFs for each PAH, and TEFs for each PAH as proposed in this study, are shown in Table 2. As toxicities for Nap, Ace, Flu, and Fla were not included in the TEF system reported by the US EPA, those missing TEF values were adopted from the work of Nisbet and LaGoy.

The total toxicity equivalency concentrations (BaPeq) were calculated using the following Equation:

∑BaPeq= ∑(C_i_ × TEF_i_) (1)

Here, ∑BaPeq is the total toxic equivalent concentration of identified PAHs, C_i_ is the concentration of individual PAHs, and TEF_i_ is the corresponding toxic equivalency factor.

**Figure 8:**
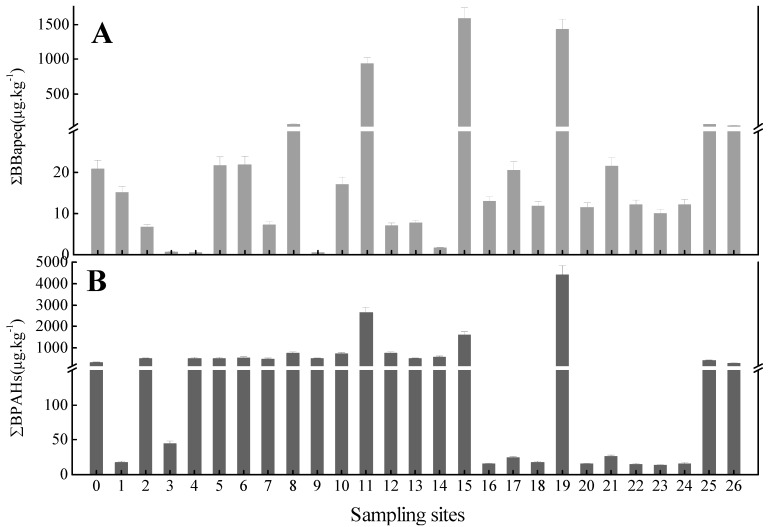
Show concentrations (∑BPAHs) and toxic equivalent concentrations (∑BBaPeq) of BPAHs in sampling sites (µg·kg^−1^). Notes: (**A**): shows the means concentrations (∑BPAHs) of BPAHs; (**B**): shows the toxic equivalent concentrations (∑BBaPeq) of ∑BPAHs; BPAHs: carcinogenic PAHs.

The concentrations of BPAHs (∑BPAHs) are shown in Figure 8B. It shows that in our soil samples, ∑BPAHs ranged from 13.3 µg·kg^−1^ to 4397 µg·kg^−1^. The toxic equivalent concentrations of BPAHs (∑BBaPeq) in all sampling sites are shown in Figure 8A. Based on carcinogenic effects and the risk posed by PAHs, soil criteria for the protection of human health have been established in Canada, at a safe ΣBaPeq level of 600.0 µg·kg^−1^ [35]. Only three sample sites (S11, S15, and S19) from the Dagang Oilfield were above this standard value. The mean ΣBaPeq of all sites was 161.2 µg·kg^−1^, with this level lower compared to that in other sites such as Shanghai (428.0 µg·kg^−1^) [28] and Beijing (181.0µg·kg^−1^) urban soils [36], but higher than in Tarragona (124.0 µg·kg^−1^) [32].Our results imply a lower risk to the productive habitat functions of sample sites, but due to different sources of PAHs, the potential hazard levels vary significantly across sample sites.

**Table 2:** Proposed TEFs for individual PAHs.

Compound	USEPA	Nisbet And LaGoy	This Study	Carcinogenicity Classification
Nap	0	0.001	0.001	B ^a^
Ace	0	0.001	0.001	
AcP	0	0.001	0.001	
Flu	0	0.001	0.001	
Phe	0	0.001	0.001	
Ant	0	0.01	0.01	
Fla	0	0.001	0.001	
Pyr	0	0.001	0.001	
Baa	1	0.1	1	B
Chr	1	0.01	1	B
Bbf	1	0.1	0.1	B
BkF	1	0.1	1	B
BaP	1	1	1	B
DahA	1	1	1	B
BghiP	0	0.01	0.01	
Inp	1	1	1	B

Note: B ^a^: Nap listed in B group, which is possibly carcinogenic to humans; B: A group of PAHs that is probably carcinogenic to humans.

## 4. Conclusions

Thirteen PAHs on the US EPA priority control list were detectable in the Dagang Oilfield. Of these, 2-ring and 3-ring PAHs were the main components of pollution, with a mean ratio of 38.4% and 27.8% to total PAH concentrations, respectively. The mean concentrations of PAHs were 919.8 μg·kg^−1^, with 22% of sampling sites described as heavily contaminated, 22% as moderately contaminated, 37% as lightly contaminated, and 19% as not contaminated. The evaluation of profiles of individual compounds indicate that PAHs are derived from two sources - petroleum deposition and anthropogenic deposition originating, mainly, from the combustion of fossil fuels such as petroleum, coal, and wood. The evaluation of spatial trends in PAH concentrations in Dagang Oilfield soil indicates that sources of PAHs relate to the utility pattern of soils. There was a trend of increasing contamination along the gradient AD-OW-RD, with oil industrial products and anthropogenic activities directly or indirectly influencing the level of PAH contamination in soils. The mean concentration of BBaPeq in sampling sites was 161.2 µg·kg^−1^, implying a very low risk to Dagang Oilfield’s habitat functions. However, there are significant differences across sampling sites (from 0.49 µg·kg^−1^ to 1586 µg·kg^−1^), suggesting some level of ecological environmental risk in polluted oilfield soil. We will plan to continue research with a further study on the biotoxicity and ecotoxicity of PAHs in the oilfield areas.
